# Effects of poultry by-products inclusion in dry food on nutrient digestibility and fecal quality in Beagle dogs

**DOI:** 10.1371/journal.pone.0276398

**Published:** 2022-11-17

**Authors:** Amr Abd El-Wahab, Anna Lisa Zeiger, Bussarakam Chuppava, Christian Visscher, Josef Kamphues

**Affiliations:** 1 Institute for Animal Nutrition, University of Veterinary Medicine Hannover, Foundation, Hannover, Germany; 2 Department of Nutrition and Nutritional Deficiency Diseases, Faculty of Veterinary Medicine, Mansoura University, Mansoura, Egypt; The University of Sydney, AUSTRALIA

## Abstract

Animal by-product meals show large variability in diet digestibility. This study aimed to provide information on including bone protein meal (BPM) or feather meal (FM) in extruded dog diets with regard to digestibility and fecal characteristics in two trials. In the first trial, compared to the control (BPM0), 6, 12, and 24% of the basic diets were replaced by BPM (BPM6, BPM12, and BPM24, respectively). In the second trial, in comparison to the control (FM0), 5, 10, and 20% of the basic diets were replaced by FM (FM5, FM10, and FM20, respectively). In both trials, six Beagle dogs (BW 17.3±2.14 and 18.1±2.04 kg for trials 1 and 2, respectively) participated in a crossover experiment design. Five days were used as wash-out before each experimental period for each trial. The fecal consistency scores were based on a 5-point scale (1 = very hard, 2 = solid, well formed “optimum”, and 5 = watery diarrhea). In the first trial, results showed that the apparent digestibility of dry matter, crude protein, and crude fat was significantly lower for dogs fed BPM6 compared to those fed BPM24. There was a lower number of dogs with a fecal consistency score value > 2 (16.7%) among those fed BPM6 (median = 2, Interquartile range (IQR) = 0) compared to those fed BPM24 (83.3%). The fecal dry matter content was significantly (*p* < 0.05) the highest (39.4%±2.15) for dogs fed BPM6. In the second trial, the data revealed that dogs fed FM0 had significantly (*p* < 0.05) the highest organic matter digestibility (87.2%±1.05), while dogs fed FM20 had significantly (*p* < 0.05) the lowest crude fat digestibility (95.0%±0.95). Inclusion of FM at 10% or 20% in the diet decreased fecal dry matter significantly (29.0%±2.10 and 27.9%±2.46, respectively) compared to those animals offered FM0 (31.1%±2.56). Among those dogs fed FM0 and FM5, there was a lower significant number of dogs with a fecal score value > 2 (16.7% and 16.7%, respectively; *p* < 0.05). While the fecal score was significantly a higher (median = 4, IQR = 0) for dogs fed FM20. Including FM at any level in the diet resulted in significantly higher levels of iso-butyric and iso-valeric acids compared to FM0. These findings in both trials suggest that apparent crude protein digestibility was not affected when diets containing BPM up to 24% and FM up to 20% were offered, but fecal quality was reduced.

## Introduction

Pet ownership is still increasing in many areas worldwide and pets are increasingly considered a member of the family [1]. Over 63 million households in the United States owned at least one dog according to a 2019/2020 pet owners survey, making them the most widely owned type of pet across the United States at this time [2]. In 2020, an estimated 88 million European households owned at least one pet; 24% of households owned dogs [3]. There were 89 million pet dogs in 2020 in Europe, showing a 20% increase compared to a total of 74 million dogs in 2010 [3]. In line with the increasing number of pet owners, the world human population is expected to increase to around 9.6 billion by the year 2050 [4]. According to current patterns, global meat consumption among humans is forecast to increase by 158 million tons by 2030, and by 233 million tons by 2050 [5]. Nonetheless, there is a significant portion of nutritionally valuable animal by-products that does not enter the human food system, and which could be offered to pets. Animal by-products are defined in Article 3 of Regulation (EC) 1069/2009 as “entire bodies or parts of animals, products of animal origin or other products obtained from animals that are not intended for human consumption” [6]. Average carcass yield, or dressing percentage, ranges between 50% and 74% of live animal weight for red meat and poultry products, respectively in the United States, resulting in a significant portion of animal-derived material that does not enter the human food system. For instance, meat and bone meal, meat meal, poultry meal, hydrolyzed feather meal (FM), blood meal, and animal fats are some of the primary products resulting from the rendering process [7, 8]. A total of 100 to 150 million tons/year of meat by-products are generated from food animal slaughter [9]. In North America, approximately 25 million tons/year of raw materials are rendered, producing about 5 million tons of fats and a similar quantity of protein meals [10]. To support a sustainable future, this massive quantity of material must be handled with methods that are safe, environmentally responsible, and efficient with respect to recovery of valuable resources [11].

To the best of our knowledge, the impact of the modern processing techniques on the nutrient profile for bone protein meal (BPM) or FM and its inclusion in dog food as a protein source is very limited. Thus, there are no sufficient data on the digestibility of a diet containing BPM or FM and its impact on fecal quality in dogs. Furthermore, including of FM may be a low-cost source of protein in poultry, pig, dog, and cat diets [12]. Although FM has a low biological value for monogastric animals, it provides the diet with some essential amino acids, such as leucine and isoleucine [13, 14]. To make the proteins available for animal digestion and absorption, feathers should be treated at a high heat and pressure to breakdown the structure of keratin [12]. However, long-term exposure of feathers to these conditions may affect the availability of amino acids negatively, especially cysteine, which is the most sensitive indicators of processing effects [15–17].

There is a growing interest in alternative protein sources in general to satisfy marketing/consumer demand or in using by-products from other industries to improve sustainability. Recently, Acuff et al. [18] defined sustainability as “The conscientious management of resources and waste necessary to meet the physiologic requirements of companion animals without compromising the ability of future generations to meet their environmental, social, or economic needs”. Considering this need, the objective of this study was to provide information on including two by-products, BPM or FM, to extruded dog diets with regard to apparent nutrient digestibility as well as fecal characteristics and fatty acids profile.

## Materials and methods

This study protocol was reviewed and approved by the Animal Welfare Officer of the University of Veterinary Medicine Hannover, Foundation, Hannover, Germany in accordance with the German protocol § 7 of the Animal Protection Law prior to conducting this study (approval number TVO-2014-V-2).

### Experimental design

**Fig 1:**
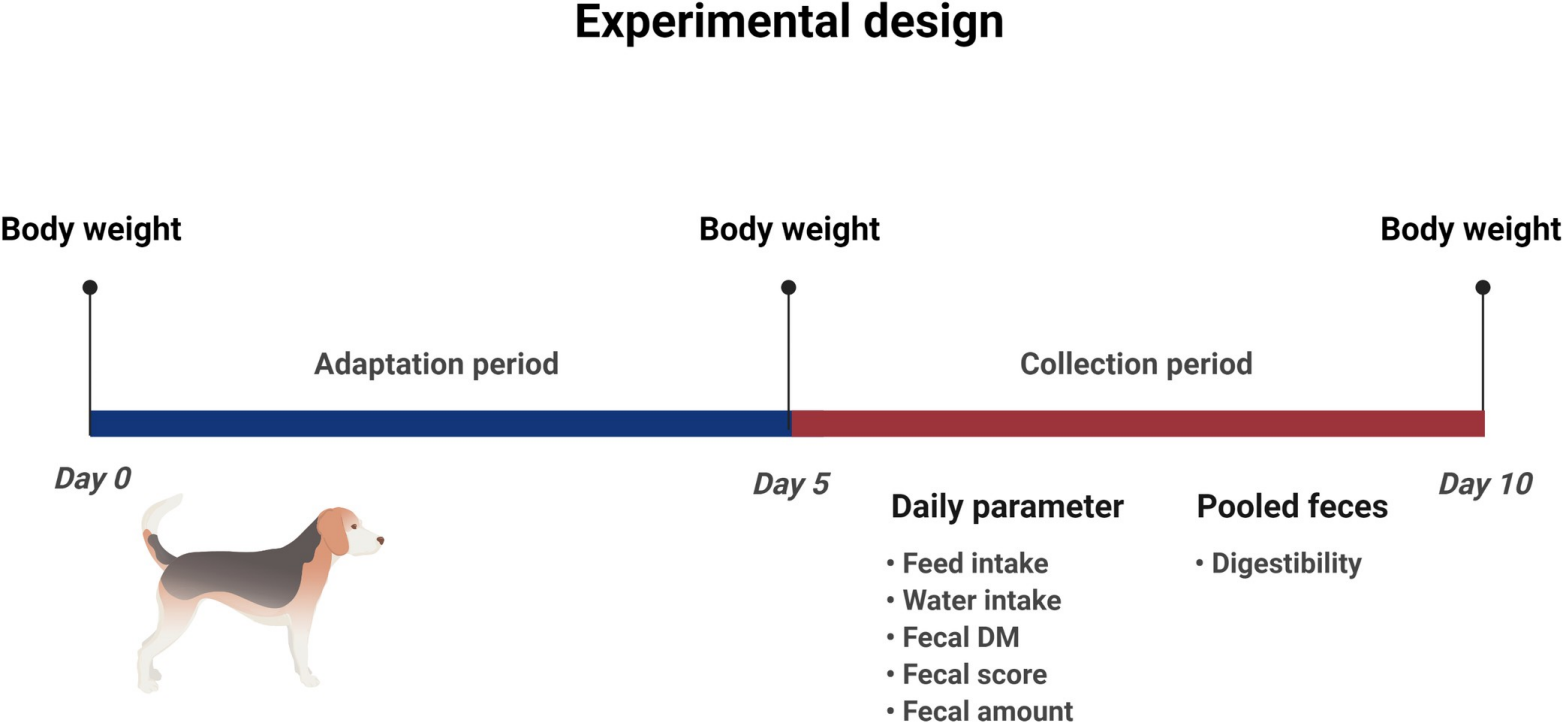
Concept of the experimental design and parameters recorded during the collecting period (figure was created with BioRender.com).

Healthy intact female Beagle dogs (*n* = 6) were included in the digestibility study at the Institute for Animal Nutrition, University of Veterinary Medicine Hannover, Foundation. All dogs included in this study derived from the University of Veterinary Medicine Hannover, Foundation. At the beginning of the study, the dogs had a mean body weight (BW) of 17.3 ± 2.14 and 18.1 ± 2.04 kg for trials 1 and 2, respectively, with ages ranging from 6 to 10 years. During the digestibility tests, dogs were housed individually in 2.00 × 3.00 m kennels to enable fecal collection. The trials were conducted using a crossover experimental design with four stages and four treatments in each trial. In each trial, the animals followed an adaptation period of 5 d, followed by 5 d of fecal collection for individual estimation of the apparent nutrient digestibility and fecal scores (Fig 1). Five days were used as wash-out before each experimental period (adaptation + collection) for each trial.

### Production of diets

#### First trial

An extruded commercial dry diet (Fit+Fun Croc, MultiFit Tiernahrungs GmbH, Krefeld, Germany) was used as a basic diet (Table 1). The BPM was submitted to 135°C and 3 bar pressure for 20 min, then pressed, grounded, and packaged. The four experimental diets were produced by replacing about 0%, 6%, 12%, and 24% of the basic diet by adding BPM (BPM0, BPM6, BPM12, and BPM24, respectively). Briefly, the dogs in the control group were fed about 350 g as feed/d of the basic diet. However for the BPM6 group (6% replacement of basic diet by BPM), the dogs were fed about 329 g as feed/d + 21 g of BPM/d. In the BPM12 and BPM24 groups (12% and 24% replacement of basic diets by BPM), the dogs were fed about 308 g as feed/d + 42 g of BPM/d and 266 g as feed/d + 84 g of BPM/d, respectively. The added amount of BPM as an ingredient (not extruded) was thoroughly mixed manually with the offered basic diet at every meal. Generally, the diet with BPM addition (i.e., simply removing a percentage of the commercial diet and adding the experimental ingredient) still satisfied the nutritional requirements in accordance with NRC (2006).

**Table 1:** Ingredients and chemical composition of the basic diet in first and second trials.

First trial^ϯ^		Second trial^§^
**Ingredient**		
Grains		Dehydrated poultry protein
Meat and animal by-products		Corn
Vegetable by-products		Rice
Oil and fat		Wheat
Minerals		Hydrolysed animal proteins
		Animal fat
		Corn gluten
		Vegetable fiber
		Vegetable protein isolate*
		Beet pulp
		Fish oil
		Minerals
**Analysed chemical composition (% DM)**		
DM (% of fresh matter)	92.4	92.0
Crude ash	8.35	6.21
Crude protein	20.7	28.0
Crude fat	7.76	20.0
Crude fiber	2.89	2.89
Nitrogen free extract	60.3	42.9
ME (MJ/100 g as feed)	1.43	1.68
Calcium	2.16	0.81
Phosphorus	1.28	0.69

^ϯ^ An extruded commercial dry diet (Fit+Fun Croc, MultiFit Tiernahrungs GmbH, Krefeld, Germany), the amount of the ingredients were not available.

^§^ An extruded commercial dry diet (Royal Canin Beagle Adult dry dog food, Crown Pet Foods Ltd., Castle Cary, UK), the amount of the ingredients were not available.

* Protein selected for its very high digestibility.

#### Second trial

In the second trial, a commercially available, extruded dry control food (Royal Canin Beagle Adult dry dog food, Crown Pet Foods Ltd., Castle Cary, UK) was used as the basic diet (Table 1). The hydrolyzed FM was produced in a conventional manner. Briefly, fresh feathers were added to the batch cooker simultaneously, followed by steam injection at 110°C for 20 min. The hydrolysis was started by raising the temperature to 160°C and pressure to 300 kPa for 40 min. Afterwards, the FM was dried for 75 min, milled through a 5 mm sieve, and bagged.

The basic diet (FM0) was supplemented with different levels of hydrolyzed FM to obtain a further three diets with 5%, 10%, and 20% FM in the basic diet (in total: FM0, FM5, FM10, and FM20). Briefly, the dogs in the control group were fed about 300 g fresh/d of the basic diet. However, in the case of the FM5 group (5% replacement of basic diet by FM), the dogs were fed about 285 g as feed/d + 15 g of FM/d. In the groups FM10 and FM20 (10% and 20% replacement of basic diets by FM), the dogs were fed about 270 g as feed/d + 30 g of FM/d and 240 g as feed/d + 60 g of FM/d, respectively. The added amount of FM as an ingredient (not extruded) was thoroughly mixed manually with the offered basic diet at every meal. Generally, the diet with FM addition still satisfied the nutritional requirements in accordance with NRC (2006).

### Chemical analysis

To determine the nutrients in the diets and fecal samples, these were analyzed using the methods of the Association of German Agricultural Analytic and Research Institutes e.V. (VDLUFA) [19]. The dry matter (DM) content was calculated by weighing the samples (about 50 g) before and after they had been dried at 103°C for 12 h. The crude ash content in the muffle furnace was determined by weighing the dried and ground samples (about 3 g) before and after combustion at 600°C for 6 h. The Dumas incineration method was also used to determine the total nitrogen content by heating about 0.3 g of the sample in a crucible at 1000°C in the Elementar analyser (Vario Max CNS, Elementar Analysensysteme GmbH, Langenfeld, Germany). The crude protein could then calculated by multiplying the nitrogen content by 6.25. The crude fat content was measured by using the Soxhlet apparatus via acid digestion. The diluted acidic and alkalic solutions and subsequent drying at 103°C (Fibertec 2010 Hot Extraktor, Foss, Sweden) were performed to measure the crude fiber content. The calcium content was determined by atomic absorption spectrometry (Solaar M-Serie Atomic Absorption Spectometer, Thermo Elemental Ltd., Cambridge, England) in accordance with Slavin [20]. A photometric characterization (UV-Visible Recording Spectrophotometer UV 162, Schimadzu, Kyoto, Japan; Wavelength 356 nm) of the phosphorus content was based on the vanadate molybdate method in accordance with Gericke and Kurmies [21]. Finally, the amino acid content was determined using ion-exchange chromatog-raphy (AA analyzer LC 3000, Biotronik Wissen-schaftliche Geräte GmbH, Maintal, Germany). The nitrogen-free extract content was calculated as follows: Dry matter—(crude ash + crude protein + crude fat + crude fiber).

### Food intake and apparent digestibility

The animals were fed once per day and received water *ad libitum*. The amount of food offered and refused was recorded after each meal-time to calculate food intake. To predict a mean of the quantity of food offered, we have first to estimate the metabolizable energy (ME) contents of the diets based on their chemical composition in accordance with Kamphues et al. [22] according to the following equation: ME (MJ/kg) = 0.01674 × crude protein + 0.03767 × crude fat + 0.1674 × nitrogen-free extract. Thereafter, the energy requirement prediction equation for adult dog following the metabolic weight an equation was used (0.5 MJ ME × BW^0.75^/d) [23]. The total fecal collection method was used to perform the apparent nutrient digestibility [24], consisting of an initial phase of 5 d of adaptation to the diet, followed by 5 d of fecal collection. During the collection period, the fresh feces were collected daily from the concrete floor. After being weighed, in a subsample of 10% of the fresh feces per animal/d, the DM content was determined. Thereafter, the remaining fecal samples were stored at –20°C. At the end of the trial, 5 d fecal samples from each dog were thawed, mixed, and homogenized. The apparent digestibility was calculated in accordance with Kamphues et al. [22] using the following formula: apparent digestibility (%) = ((food-feces)/food) × 100.

### Fecal quality

The number of defecations per day was recorded. During five consecutive days (last five days of the ten days period), the feces were collected completely and individually every 15 min maximum. In accordance with Moxham [25], the fecal consistency scores were evaluated always by the same person and calculated for each defecation using a five-point scale (1 = very hard; 2 = solid, well formed “optimum”; 3 = soft, still formed; 4 = pasty, slushy; 5 = watery diarrhea). A graphic representation of the fecal scoring system was previously described by Abd El-Wahab et al. [26]. A score of 2 represented a solid, well-formed stool at the fecal surface; this was considered optimal [27].

### pH value

To measure daily pH in fresh feces, the collected samples were mixed with distilled water at a ratio of 1:5, shaken, left at room temperature for 1 min, and subsequently measured with a pH meter (InLab^®^ Expert Pro, Mettler-Toledo International Inc., Columbus, OH, USA).

### Volatile fatty acids

On the last day of the collection phase, the fresh feces were taken from each animal to determine the fatty acids in accordance with Bunte et al. [28] by using a gas chromatography (610 Series, Unicam Chromatography GmbH & Co. KG, Kassel, Germany) with a column temperature of 155°C (injector: 175°C, detector: 180°C). The carrier gas was nitrogen, the flow rate was about 0.97 mL/min, and the detector type was flame ionization in the gas chromatography.

### Statistical analysis

The statistical analysis was performed using the Statistical Analysis System for Windows, SAS^®^ Enterprise Guide^®^, version 9.3 (SAS Institute, Inc., Cary, NC, USA). For all parameters, mean values as well as the standard deviation (SD) of the mean were calculated. While, values in the form of a score, i.e., fecal consistency score; median values and interquartile range (IQR) were determined. All measured or recorded parameters were analyzed individually and formed the basis of the calculation.

Depending on the distribution analysis of the data, both parametric and non-parametric methods were applied. To compare mean values of the apparent nutrient digestibility, fecal DM content, fecal pH as well as the fatty acid profile in the feces, the normal distribution of the residuals was first tested using a Shapiro-Wilk test. Differences among treatments were determined using a multi-range test (Ryan-Einot-Gabriel-Welsch test). Linear and quadratic effects were tested using orthogonal polynomial contrasts. For non-normally distributed data, e.g., fecal defecation frequency or values in the form of a score, the Kruskal-Wallis test was used.

Values in the form of a fecal consistency score, two-dimensional frequency distributions of categorical features were checked for dependency using Pearson’s chi-square test of homogeneity, provided the sample was evenly distributed. Otherwise, Fisher’s exact test was used. The significance level was determined at *p* < 0.05.

## Results

The general condition of dogs was healthy throughout the experimental period. The median body condition score during the whole experimental trial was 5 out of 9 in accordance with Laflamme [29]. No refusals were observed in either of the trials. All dogs consumed the total amount of the daily food offered to all groups (350 g as fed/dog in the first trial and 300 g as fed/dog in the second trial). In both trials, the BW of the dogs was similar among the groups at the beginning of the study (*p* > 0.05) and did not change throughout the study (*p* > 0.05).

### Chemical composition of experimental diets

#### First trial

The amino acids profile of the BPM as an ingredient is presented in Table 2.

**Table 2:** Levels of amino acids in the experimental raw ingredients (g/kg DM).

Amino acid	BPM	FM
Asparagine	47.6	61.8
Threonine	20.7	39.9
Serine	21.1	103
Glutamine	83.9	103
Glycine	67.1	72.3
Alanine	41.5	46.0
Valine	23.2	70.6
Cysteine	4.12	42.9
Methionine	11.5	5.45
Isoleucine	20.1	49.0
Leucine	36.9	76.1
Tyrosine	13.3	28.5
Phenylalanine	19.8	48.8
Histidine	12.3	7.38
Lysine	35.6	19.4
Arginine	40.6	67.1
Proline	46.7	104

BPM = Bone protein meal as an ingredient.

FM = Feather meal as an ingredient.

The chemical composition of the experimental diets (Table 3) in this study varied considerably due to different levels of the BPM ingredient profiles. The DM content between the experimental canine food was virtually similar (range: 92.4–93.3% of fresh matter). The crude ash, crude protein, and crude fat contents were increased linearly with an increasing inclusion level of BPM, while the level of crude fiber decreased with an increasing inclusion level of BPM.

**Table 3:** Chemical composition of the ingredient and basic diets supplemented due to different levels of BPM.

Item	Unit	Ingredient	Experimental diets			
		BPM	BPM0	BPM6	BPM12	BPM24
Basic diet	%	-	100	94	88	76
BPM			0	6	12	24
DM	% of fresh matter	96.3	92.4	92.6	92.9	93.3
Crude ash	% DM	18.6	8.35	8.97	9.58	10.8
Crude protein		65.8	20.7	23.4	26.1	31.5
Crude fat		14.5	7.78	8.18	8.59	9.39
Crude fiber		0.00	2.89	2.72	2.54	2.20
Nitrogen free extract		1.10	60.3	56.7	53.2	46.1
Calcium		0.53	2.16	2.35	2.54	2.92
Phosphorus		0.31	1.28	1.39	1.50	1.72
ME	MJ/100 g	18.3	1.43	1.44	1.45	1.48
	as fed					

BPM = Bone protein meal as an ingredient.

Sums of crude ash, crude fat, crude protein, crude fiber, and nitrogen-free extracts may not total 1000 g due to rounding up.

#### Second trial

The amino acids profile of the FM as an ingredient is presented in Table 2. The chemical composition of the experimental diets (Table 4) in this study showed some differences in the chemical analyses. The DM content between the experimental canine foods was similar (range: 92.0–92.4%). Only the crude protein content was increased linearly with an increasing level of FM inclusion, whereas the levels of crude ash, crude fat, and crude fiber decreased with an increasing inclusion level of FM.

**Table 4:** Chemical composition of the ingredients and basic diets supplemented due to different levels of FM.

Item	Unit	Ingredient	Experimental diets			
		FM	FM0	FM5	FM10	FM 20
Basic diet	%	-	100	95	90	80
FM			0	5	10	20
DM	% of fresh matter	94.1	92.0	92.1	92.2	92.4
Crude ash	% DM	1.76	6.21	5.99	5.77	5.32
Crude protein		93.1	28.0	31.3	34.5	41.0
Crude fat		0.65	20.0	19.3	18.7	17.3
Crude fiber		0.00	2.89	2.7.	2.60	2.31
Nitrogen free extract		0.00	42.9	40.8	38.6	34.3
Calcium		0.45	0.81	0.79	0.77	0.74
Phosphorus		0.66	0.69	0.67	0.64	0.60
ME	MJ/100 g	18.0	1.68	1.68	1.69	1.69
	as fed					

FM = Feather meal as an ingredient.

Sums of crude ash, crude fat, crude protein, crude fiber, and nitrogen-free extracts may not total 1000 g due to rounding up.

### Apparent nutrient digestibility

The results of the apparent nutrient digestibility are presented in Tables 5 and 6. In the first trial (Table 5), the organic matter digestibility varied only significantly between dogs fed either BPM6 or BPM12 (80.4%±2.23 vs 82.8%±1.07). Moreover, increasing the inclusion of BPM up to 24% exhibited a linear increase in the digestibility of crude protein (*p* = 0.034) and crude fat (*p* = 0.003). The apparent digestibility for crude protein was significantly higher (82.4%±1.22) for dogs fed BPM24 compared to those fed BPM0 (78.0%±4.40) or BPM6 (76.3%±2.93). Besides, dogs fed BPM24 showed significantly higher crude fat digestibility (89.5%±1.15) in comparison to those fed BPM0 and BPM6 diets (86.0%±2.65, and 85.4%±2.13, respectively).

**Table 5:** Apparent nutrient digestibility (%) and fecal characteristics of dogs fed basic diets supplemented with different levels of BPM in first trial (mean±SD).

Parameters	Experimental diets				*p*-value	*p*-value	
	BPM0	BPM6	BPM12	BPM24		Linear	Quadratic
**Apparent nutrient digestibility**							
Dry matter	76.9^a^±3.35	73.7^b^±2.99	77.5^a^±1.04	77.6^a^±1.47	0.039	0.238	0.168
Organic matter	81.4^ab^±2.97	80.4^b^±2.23	82.8^a^±1.07	81.5^ab^±1.23	0.046	0.168	0.306
Crude protein	78.0^b^±4.40	76.3^b^±2.93	79.4^ab^±2.20	82.4^a^±1.22	0.030	0.034	0.194
Crude fat	86.0^bc^±2.65	85.4^c^±2.13	88.0^ab^±0.26	89.5^a^±1.15	0.002	0.003	0.173
**Fecal characteristics**							
Defecation frequency (n/d)	2.43^ab^±0.63	2.70^a^±0.55	2.37^ab^±0.54	2.30^b^±0.60	0.036	0.264	0.660
Fecal consistency score with a score value > 2 (%)^1^	83.3	16.7	66.7	83.3	<0.050	-	-
Fecal consistency score^1^^,^*	3 (0)	2 (0)	3 (1)	3 (1)	<0.050	-	-
Amount of feces (g DM/d)	74.7±10.8	85.3±9.76	73.0±3.31	73.7±4.94	0.656	0.185	0.674
DM content (%)	31.5^b^±1.38	39.4^a^±2.15	31.6^b^±2.71	32.1^b^±2.30	0.001	0.230	0.553
pH value	6.92±0.14	6.76±0.18	6.77±0.28	6.90±0.21	0.449	0.164	0.351

BPM = Bone protein meal.

^a,b,c^ Means in a row with different superscripts differ significantly (*p* < 0.05).

^1^Fecal scores were recorded using a five-point scale (1 = very hard to 5 = watery diarrhea) and score of 2 was considered optimal.

* Fecal consistency score results are presented in median values (Interquartile range).

**Table 6:** Apparent nutrient digestibility (%) and fecal characteristics of dogs fed basic diets supplemented with different levels of FM in second trial (mean±SD).

Parameters	Experimental diets				*p*-value	*p*-value	
	FM0	FM5	FM10	FM20		Linear	Quadratic
**Apparent nutrient digestibility**							
Dry matter	83.5±1.11	81.9±2.62	82.4±1.69	84.4±5.27	0.526	0.583	0.070
Organic matter	87.2^a^±1.05	85.7^b^±2.19	85.6^b^±1.60	85.5^b^±2.62	0.047	0.407	0.403
Crude protein	82.4±1.67	80.8±3.23	80.5±3.21	81.1±3.74	0.732	0.359	0.584
Crude fat	96.5^a^±0.84	96.0^a^±1.20	96.2^a^±0.62	95.0^b^±0.95	0.046	0.106	.0.433
**Fecal characteristics**							
Defecation frequency (n/d)	1.87^a^±0.30	1.83^ab^±0.51	1.53^bc^±0.24	1.47^c^±0.30	0.038	0.001	0.771
Fecal consistency score with a score value > 2 (%)^1^	16.7	16.7	83.3	100	<0.050	-	-
Fecal consistency score^1^^,^*	2 (0)	3 (1)	3 (1)	4 (0)	<0.050	-	-
Amount of feces (g DM/d)	45.5^b^±3.13	50.5^a^±8.06	48.6^ab^±4.68	48.0^ab^±7.84	0.019	0.537	0.602
DM content (%)	31.1^a^±2.56	30.8^a^±1.83	29.0^b^±2.10	27.9^b^±2.46	0.037	0.001	0.761
pH value	6.39^b^±0.14	6.40^b^±0.22	6.42^b^±0.16	6.62^a^±0.06	0.041	0.051	0.165

FM = Feather meal.

^a,b,c^ Means in a row with different superscripts differ significantly (*p* < 0.05).

^1^Fecal scores were recorded using a five-point scale (1 = very hard to 5 = watery diarrhea) and score of 2 was considered optimal.

* Fecal consistency score results are presented in median values (Interquartile range).

Regarding the second trial (Table 6), no significant differences were observed for dry matter and crude protein apparent digestibility among treatments, while dogs fed FM0 had the highest organic matter digestibility (87.2%±1.05) compared to other treatments. The crude fat digestibility was significantly the lowest (95.0%±0.95) for dogs fed FM20 in comparison to other treatments.

### Fecal quality

In the first trial (Table 5), when evaluating the daily defecation frequency during the collection period, the average number of defecations per day was significantly higher for dogs fed BPM6 (2.70 times/day, median = 3, Interquartile range (IQR) = 1) compared to those fed BPM24 (2.30 times/day, median = 2, IQR = 1). Fecal scores showed significant differences among treatments (*p* < 0.05). The fecal consistency score value > 2 (score of 2 was considered optimal) was significantly higher in dogs fed BPM0, BPM12 and BPM24 (83.3%, 66.7% and 83.3%, respectively). Dogs fed BPM6 showed a lower fecal score (median = 2, IQR = 0) and increased with increasing BPM inclusion in the diets (median = 3, IQR = 1 and median = 3, IQR = 1 for BPM12 and BPM24, respectively). However, no significant differences in fecal DM content (range: 31.5%-32.1%) were seen among treatments for dogs fed BPM0, BPM12, and BPM24 diets, while dogs fed the BPM6 diet had significantly the highest fecal DM content (39.4%±2.15). No significant differences were noted in the fecal pH values among the treatments.

In the second trial (Table 6), increasing the inclusion of FM up to 20% (FM20) exhibited a linear decrease in the defecation frequency (1.47 times/day, median = 1.5, IQR = 1) compared to FM0 (1.87 times/day, median = 2, IQR = 0). When evaluating the fecal consistency score, there was a high significant number with a fecal score value > 2 (100% and 83.3%, respectively) among those dogs fed FM20 and FM10, while dogs fed FM5 and FM0 had a lower number with a fecal score value > 2 (16.7% and 16.7%, respectively). Dogs fed FM0 showed a lower fecal score (median = 2, IQR = 0), which increased with increasing FM inclusion in the diets (median = 3, IQR = 1 for FM6, median = 3, IQR = 1 for FM12, and median = 4, IQR = 0 for FM24, respectively). Fecal DM was greatest for the control treatment (FM0) and decreased (*p* = 0.001) linearly with increasing FM inclusion in the diets. Dogs fed FM10 and FM20 had significantly lower fecal DM content (29.0%±2.10 and 27.9%±2.46, respectively) compared to those fed FM0 and FM5 (31.1%±2.56 and 30.8%±1.83, respectively). Dogs fed FM20 had significantly the highest fecal pH value (6.62±0.06) compared to other treatments (range: 6.39–6.42).

### Volatile fatty acids

Data from the fatty acid pattern in the feces of dogs fed different experimental diets in both trials are presented in Tables 7 and 8. In the first trial with BPM supplementation in the diet, there were no significant differences in the fecal fatty acids profile among treatments (Table 7).

**Table 7:** Fatty acid profile in the feces (mmol/kg fresh feces) of dogs fed the basic diets supplemented with different levels of BPM in first trial (mean±SD).

Parameters	Experimental diets				*p*-value	*p*-value	
	BPM0	BPM6	BPM12	BPM24		Linear	Quadratic
acetic acid	29.7±8.91	28.6±14.3	25.3±11.7	38.4±18.4	0.417	0.101	0.373
propionic acid	13.7±4.69	15.4±9.06	31.8±50.6	18.4±8.61	0.629	0.251	0.232
iso-butyric acid	0.68±0.29	0.58±0.15	0.65±0.36	1.01±0.33	0.089	0.079	0.527
n-butyric acid	11.6±9.93	7.94±3.46	7.51±5.66	9.63±4.40	0.069	0.112	0.067
iso-valeric acid	1.04±0.47	0.81±0.21	0.89±0.44	1.45±0.52	0.068	0.054	0.373
n-valeric acid	0.14±0.16	0.12±0.09	0.11±0.08	0.12±0.09	0.958	0.115	0.061

BPM = Bone protein meal.

No significant differences were noted among treatments, so that no superscripts were added.

**Table 8:** Fatty acid profile in feces (mmol/kg fresh feces) of dogs fed the basic diets supplemented with different levels of FM in second trial (mean±SD).

Parameters	Experimental diets				*p*-value	*p*-value	
	FM0	FM5	FM10	FM20		Linear	Quadratic
acetic acid	35.3±17.3	54.1±11.2	37.4±14.7	39.9±7.77	0.092	0.114	0.198
propionic acid	16.1^b^±6.29	28.5^a^±4.52	16.0^b^±3.74	17.0^b^±5.22	0.001	0.210	0.437
iso-butyric acid	0.52^b^±0.20	1.64^a^±0.67	1.47^a^±0.44	1.93^a^±0.48	0.001	0.079	0.328
n-butyric acid	10.5±9.18	24.0±16.7	14.5±9.88	10.9±5.81	0.160	0.251	0.056
iso-valeric acid	0.79^b^±0.33	2.34^a^±0.91	2.11^a^±0.76	2.55^a^±0.90	0.003	0.055	0.323
n-valeric acid	0.09±0.14	0.27±0.16	0.17±0.16	0.17±0.09	0.230	0.086	0.744

FM = Feather meal.

^a,b^ Means in a row with different superscripts differ significantly (*p* < 0.05).

In the second trial (Table 8), a significant increase in the propionic acid content was observed with an increasing inclusion level of FM up to 5% (28.5 mmol/kg) compared to other treatments (range: 16.0–17.0 mmol/kg). Dogs offered FM up to 20% had the highest significant increase (range: 1.47–1.93 mmol/kg) in the iso-butyric acid content compared to the control diet (0.52 mmol/kg). Similarly, dogs fed FM up to 20% had the highest significant increase (range: 2.11–2.55 mmol/kg) in the iso-valeric acid content compared to the control diet (0.79 mmol/kg).

## Discussion

In the first trial, it seemed that including BPM (regardless of inclusion level) led to a similar organic matter digestibility compared to those dogs fed BPM0 (range: 80.4%-82.8% for BPM diets vs 81.4% for BPM0). In the second trial, the apparent digestibility of organic matter was negatively affected by the inclusion of FM regardless of its level in comparison to those dogs fed the FM0 diet (range 85.5%-85.7% for FM diets vs 87.2% for FM0). This is in accordance with Zentek [30] and Bosch et al. [31] who stated that the apparent digestibility of organic matter was influenced by the amount of dietary protein. Apart from that, the inter-chain disulfide bonds in the keratin structure result in low solubility of FM and great resistance to digestive enzymes, such as pepsin and trypsin, in monogastric animals [32], which could affect the organic matter digestibility.

Whereas including BPM up to 24% increased the protein digestibility (82.4%), particularly when compared to BPM0 (78.0%), in the second trial, however, the effect of including FM had no effect on protein digestibility. Although protein adequacy requires the correct amino acids to be absorbed in their appropriate concentrations, the protein digestibility is a valuable indicator of protein quality [33]. A significant observation from the data in our study is that the food tested here had a protein digestibility that varied within the normal digestibility (80%) described by the European Pet Food Industry Federation (FEDIAF) [34].

The digestibility of BPM and FM probably depends on the composition and quality of the components, but also on the processing thereof. This may be related in part to the proper processing of the ingredients and the food, since the thermal process inactivates the anti-nutritional factors present in the meal, such as keratin [32]. Additionally, one factor that could affect protein digestibility is dietary crude ash content. Despite the content of dietary crude ash being raised when increasing the inclusion of BPM, the apparent digestibility of crude protein went up. Contrary to our findings, Meyer and Mundt [35] stated that a higher crude ash content in the food possibly leads to insufficient acidification of the chyme, which may result in lower protein digestibility. Interestingly, in the second trial, despite the content of dietary crude ash being slightly decreased when increasing the inclusion of FM (62.1 vs 53.2 g/kg DM for FM0 and FM20, respectively), the apparent digestibility of crude protein was unaffected (82.4% for FM0 vs 81.1% FM20). The processing technique of hydrolysis of FM seems to be a reason for this.

The nutritional composition and nutrient availability of FM can vary widely between diets available on the market, and this variability may be related to the type of processing [36]. The most economical method for hydrolysis of feathers is that of using high temperature and pressure which acts on the disulfide bonds, allowing proteolytic digestive enzymes to act on the keratin in the gastrointestinal tract with improvement in amino acid availability compared to that for fresh feathers [37]. Moreover, protein denaturation by high temperatures causes complexation or destruction of thermosensitive amino acids, especially cysteine [37] and the formation of nutrients such as lysinoalanine and lanthionine [38, 39], resulting in ingredients with high levels of crude protein, but poor amino acid bioavailability.

In the present study, the type of diet offered to dogs affected the apparent fat digestibility. The fat content increased with inclusion of BPM in the diet, and the fat digestibility was also increased in BPM20 to about 89.5% vs 86.0 for BPM0. In the second trial, dogs fed FM20 diets containing lower fat content when compared with FM0, resulted in crude fat digestibility of about 95% for FM20 vs 96.5% for FM0. The low-fat digestibility for BPM diets could be due to low fat content in these diets. It has been already acknowledged that fat diet digestibility may increase when the content of dietary fat increases [40]. Further studies are still needed to investigate the low-fat digestibility (89%) in the current study. Hill et al. [40] noted that the digestibility of fat reached about 99% when dogs were offered diets containing a high amount of fat (320 g/kg DM). Zuo et al. [41] found that fat digestibility increased to 97% when the amount of dietary fat increased. The fat in FM consists of oil, which is considered one of the most digestible fats employed in poultry feed [42]. Freudenthal [43], on the other hand, found that fat digestibility increased with a higher proportion of unsaturated fatty acids. However, in our study, we did not measure the content of unsaturated fatty acids. Overall, and based on the data in our study, the content of crude fat in dog food appears to have contributed to crude fat digestibility.

In the current study, a high number of dogs with a fecal consistency score closer to the optimal value (score 2) was observed among dogs fed BPM6 (with a median = 2 and IQR = 0) when compared with other treatments. Thus, a clearly negative influence of very high BPM inclusion (up to 24%; median = 3 and IQR = 1) on fecal quality could be demonstrated. In the second trial, the increasing FM content of 20% in the diet seems to have adversely affected the fecal consistency score (with median = 4 and IQR = 0 for FM20) when compared with dogs fed FM0 diets (median = 2, IQR = 0). In the current study, the crude protein content in the BPM24 diet was 81 g/kg DM higher than that found in the BPM6. Also, in the second trial, the crude protein content in the FM20 diet was 97 g/kg DM higher than that found in the FM5. Nery et al. [44] observed a softer fecal consistency at higher protein levels (39% vs 21.5%) in canine food and explained this by increased fermentative degradation in the colon as the contents of ammonia, branched-chain and short-chain fatty acids in the feces were significantly increased. Opposing results were reported by Pacheco et al. [12] who found that inclusion of 7% and 15% of hydrolyzed FM produced feces lying within an appropriate fecal score.

Notably, in the current study, offering either BPM6 or FM5 diets to dogs resulted in significantly higher fecal DM content (39.4%, and 30.8%, respectively) compared to BPM24 or FM20. There are many variables that can affect fecal quality, including nutrient digestibility, food intake, and composition on the microbial activity in the gastro-intestinal tract of dogs [45, 46]. Protein digestion and absorption are considered to be one of the dietary factors affecting fecal DM content [47]. If protein is present but not absorbed, the dietary amino acids in that protein are not available for the dogs, and provide nitrogen substrate for proteolytic bacteria, which may result in reduced fecal quality [48]. This is not in agreement with our findings, offering a low inclusion of BPM in the diet decreased an apparent digestibility of crude protein but the fecal quality was still good compared to those offered a high inclusion of BPM. In the present study, a higher water content in the feces of dogs fed with FM10, and FM20 diets was probably related to the higher flow of undigested proteins in the large intestine, due to the higher protein content in these diets [49]. However, the result on the effects of including of BPM or FM in the dog food on fecal quality is still not clear, so that other factors should be considered, such as the fecal microbiota of dogs are still needed to determine the effect of BPM or FM.

Remarkably, in the current study, offering up to 24% BPM in the dog food did not affect the fecal fatty acids profile. However, inclusion of FM resulted in increased levels of iso-butyric acid and iso-valeric acid. The production of different proportions of short-chain fatty acids and branched-chain fatty acids can also be influenced by the availability of fermentable carbohydrates and non-digestible proteins. Fermentation of soluble fibre is accompanied by the production of short chain fatty acids such as acetic, propionic, and butyric acid. The positive effects of butyrate can be achieved by adding fermentable fibre to the diet. However fibre may also interact with nutrient digestibility and availability [50] and too large amounts of fermentable fibre can induce negative fecal characteristics such as loose stools [51]. Overdosing of butyrate might induce an osmotic effect resulting in increased faecal moisture content and worse faecal consistency [23]. Short-chain fatty acids are mainly a result of amino acids and/or carbohydrate fermentation, whereas branched-chain fatty acids result from the fermentation of branched-chain amino acids [52]. Iso-butyrate and isovalerate are produced from valine and leucine, respectively [53]. It should be noted that the isovalerate peak from gas chromatography analysis also includes 2-methylbutyrate, which would be produced from fermentation of isoleucine [54]. Possibly iso-butyrate is the branched chain fatty acids with the most importance, because of its similarities with butyrate [55]. The important aspect of the fermentation is the rate of production of these products. Additionally, the increase in concentration of fermentation end products could shift the osmotic balance in the colon and favor water and sodium transport toward the lumen. Thus, feeding a diet rich in soluble and rapidly fermentable fiber could lead to flatulence, diarrhea and hence affect nutrient utilization [56].

## Conclusions

The sustainability of food animal production is greatly enhanced by recycling animal by-products like bone protein meal and feather meal during the rendering process and using those by-products as feed ingredients for companion animals. In the current study, it was observed that including either BPM or FM in the dog diets was well accepted and dogs consumed the total amount of the food offered. Apparent protein digestibility was not affected when diets containing BPM up to 24% and FM up to 20% were offered, but fecal quality was reduced and limited the use of these meals. However, it was possible to include BPM up to 12% and FM up to 5% without these having any negative effects on the fecal scores. Finally, it has to be mentioned that only female dogs were included in the current study, which may affect the digestibility data. Thus, future studies are still needed in order to remove the effect of sex by equally and randomly assigning males and females to the groups.
